# Enhanced luminescence/photodetecting bifunctional devices based on ZnO:Ga microwire/p-Si heterojunction by incorporating Ag nanowires

**DOI:** 10.1039/d1na00428j

**Published:** 2021-08-09

**Authors:** Yang Liu, Ruiming Dai, Mingming Jiang, Kai Tang, Peng Wan, Caixia Kan

**Affiliations:** College of Science, MIIT Key Laboratory of Aerospace Information Materials and Physics, Key Laboratory for Intelligent Nano Materials and Devices, Nanjing University of Aeronautics and Astronautics No. 29 Jiangjun Road Nanjing 211106 P. R. China mmjiang@nuaa.edu.cn cxkan@nuaa.edu.cn

## Abstract

With the disadvantages of indirect band gap, low carrier mobility, and large lattice mismatch with other semiconductor materials, one of the current challenges in Si-based materials and structures is to prepare low-dimensional high-performance optoelectronic devices. In this work, an individual ZnO microwire *via* Ga-incorproration (ZnO:Ga MW) was employed to prepare a light-emitting/detecting bifunctional heterojunction structure, combined with p-type Si crystal wafer as a hole transporting layer. In a forward-bias regime, red luminescence peaking at around 680 nm was captured. While, the fabricated heterojunction device also exhibited an obvious photoresponse in the ultraviolet wavelengths. Interestingly, the introduction of Ag nanowires (AgNWs) are utilized to increase light output with amplitude 4 times higher than with that of naked wire-based LEDs. Similarly, the performance parameters of the fabricated n-AgNWs@ZnO:Ga MW/p-Si heterojunction photodetector are significantly enhanced, containing a responsivity of 5.52 A W^−1^, detectivity of 2.34 × 10^12^ Jones, external quantum efficiency of 1.9 × 10^3^% illuminated under 370 nm at −1 V. We compare this work with previous reported photodetectors based on various ZnO/Si-based materials and structures, some performance parameters are not superior, but our constructed n-AgNWs@ZnO:Ga MW/p-Si heterojunction photodetector has comparable overall characteristics, and our findings stand out especially for providing an inexpensive and suitable pathway for developing low-cost, miniaturized and integrated ultraviolet photodetectors. The demonstration of AgNWs enhanced low-dimensional light-emitting/detecting bifunctional photodiodes can offer a promising scheme to construct high-performance Si-based optoelectronic devices.

## Abstract 2

A fabricated n-AgNWs@ZnO:Ga microwire/p-Si heterojunction is used to achieve high-performance light-emitting/detecting bifunctional devices.

## Graphical abstract

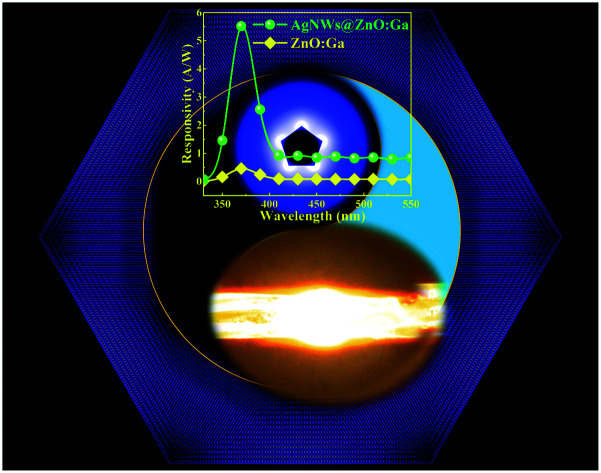

## 1 Introduction

Si-based materials and devices are one of the basic building blocks in semiconductor electronics technology in the last half century, and have attracted extensive attention for use in solar cells, light-emitting sources, photodiodes and so on.^1–7^ Due to the low-cost, high-abundance and desired electrical properties, Si-based structures are crucial to meet the needs of modern communication, processors, integrated circuits, *etc.*^8–10^ However, limited by the low carrier mobility, indirect band structure and the insulating layer around the surfaces, one of the crucial challenges in Si-based optoelectronic devices is developing power-efficient, high-speed, integrated, and smart optoelectronic devices.^11–14^ Currently, great efforts have been conducted to improve the optical and electrical properties of Si-based structures. For example, researchers have applied nano-/micro-sized Si materials to fabricate devices, constructed p–i–n structured light-emitting and photodetecting diodes, or produced a Si_*x*_M_1−*x*_ structure by incorporating extra elements (M) to modulate the band structures, *etc.*^3,15,16^ Disadvantages include the complex preparation process, stringent requirements for the equipment, high cost, and it is difficult to achieve high-performance Si-based devices, especially for light-emitting diodes (LEDs) and photodiodes. Therefore, with the development of optical communications and integrated circuits, high-performance, miniaturized, and multifunctional Si-based optoelectronic devices are highly desired and worthy of investigation.^17–20^

Profiting from its direct band structure, excellent optoelectronic properties, and controlled fabrication routes, ZnO (bandgap ∼3.37 eV, and large excitation binding energy ∼60 eV at room temperature), has attracted tremendous potential as a candidate for preparing high-performance heterostructural optoelectronic devices by combing it with other p-type semiconductor materials.^13,21–25^ Currently, rapid advances in ZnO/Si-based heterojunctions have made it possible to modulate optoelectronic performance, and great efforts have been made to fabricate ZnO/Si optoelectronic devices, like light sources, photodiodes, and solar cells, *etc.* Because of the limitation of Si materials and technology, it remains a big challenge to develop high-performance Si-based optoelectronic devices, especially for high-efficiency LEDs and photodetectors.^9,26–28^ With the development of nanotechnology, nanostructured metals with controlled morphology, components and sizes have been synthesized.^29–31^ And the uniformed nanostructures have been utilized to modulate the performances of semiconductor optoelectronic devices.^32,33^ Due to its excellent conduction characteristics, ultraviolet resonant wavelength, and high transparency, Ag nanowires (AgNWs) have been widely employed to fabricate high-performance optical and electrical structures.^34–37^

In this study, p–n heterojunctions composed of an individual ZnO microwire and Ga-doped ZnO (ZnO:Ga MW), and a p-type Si crystal wafer have been constructed. When operated in the forward-bias regime, bright luminescence peaking at around 680 nm was achieved. Meanwhile, the n-ZnO:Ga MW/p-Si heterojunction structure also exhibited an ultraviolet photoresponse when exposed to 370 nm illumination. In particular, the incorporation of AgNWs deposited on ZnO:Ga wires, can enhance the optoelectronic performance of fabricated heterojunction devices. In comparison with a single naked wire based heterostructure, an obviously enhanced light output has been achieved. Meanwhile, the incorporation of AgNWs could further be used to enhance the performance parameters of the fabricated n-AgNWs@ZnO:Ga MW/p-Si heterojunction photodetector, including response of 5.52 A W^−1^, detection of 2.34 × 10^12^ Jones, external quantum efficiency of 1.9 × 10^3^%, upon 370 nm light irradiation at −1 V. Besides, the rise and fall times of AgNWs decorating the hybrid system were also significantly decreased, and were extracted to be 362 μs and 394 μs, respectively. All of the device parameters signified that the fabricated n-AgNWs@ZnO:Ga MW/p-Si heterojunction photodetector is highly suited for practical applications of ultraviolet photodetection. Therefore, the fabricated n-AgNWs@ZnO:Ga MW/p-Si heterojunction can be used to develop high-performance light-emitting/detecting bifunctional devices. This study can deepen and promote further research and application in developing high-performance, low-dimensional Si-based optoelectronic devices.

## 2 Experimental section

### 2.1 Fabrication of an individual AgNWs@ZnO:Ga MW

**Fig. 1:**
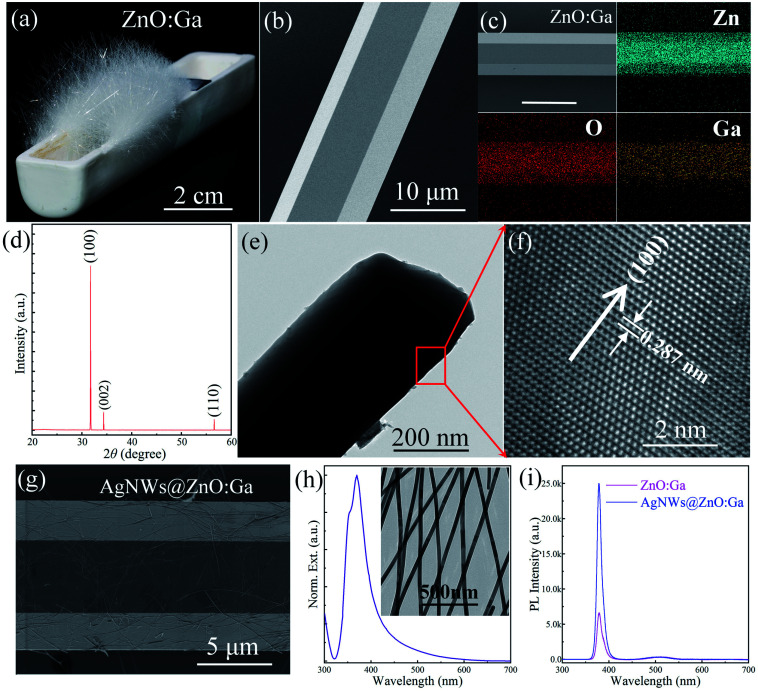
(a) Optical image of the as-grown ZnO:Ga MWs. (b) SEM image of an individual ZnO:Ga MW. (c) SEM image of an individual ZnO:Ga MW, and the corresponding EDS mapping of the uniform distribution of Zn, O, and Ga elements. The scale bar is 10 μm. (d) XRD pattern of the as-fabricated ZnO:Ga MWs. (e) TEM image of an individual ZnO:Ga MW with a width of around 350 nm. (f) High-resolution TEM image of an area of the as-fabricated ZnO:Ga wire as shown in (e). (g) SEM image of an individual ZnO:Ga wire covered by AgNWs. (h) Normalized extinction spectrum of the as-fabricated AgNWs, with the main extinction wavelength peaks at about 370 nm. Inset: TEM image of the AgNWs. (i) PL spectra of an individual ZnO:Ga wire with/without AgNWs.

As previously reported, individual ZnO:Ga MWs were synthesized with the vapor transport deposition method.^22,38^ High-purity powders of ZnO, Ga_2_O_3_ and graphite (C) with the weight ratio of 9 : 1 : 10 serving as the precursors, were placed in a corundum boat (20 cm (length) × 2 cm (width) × 1.5 cm (depth)). A cleaned Si crystal wafer (without any catalyst coating, 2 cm (length) × 2 cm (width)) was placed on the top of the mixture to collect the products. During the growth process, a constant flow of argon (Ar) (99.99%, 120 sccm) was introduced into the tube furnace as the protecting gas. The furnace temperature was firstly increased to 1100 °C in advance; then, the precursor mixture was placed in the hottest zone. Keeping the reduced oxygen synthesis conditions for about 1 hour, 10% oxygen (O_2_) was introduced into the furnace chamber as the growth gas. The as-synthesized individual ZnO:Ga MWs can be collected around the Si crystal wafer, as shown in Fig. 1(a). AgNWs were synthesized by a modified polyol method.^39,40^ The pre-synthesized AgNWs were washed using acetone and deionized water to remove the Ag nanoparticles and the residual organics. Thus, the aqueous solution containing AgNWs was successfully prepared. Using a spin-coating technique, the fabrication of an individual ZnO:Ga MW decorated with AgNWs (AgNWs@ZnO:Ga) is summarized as follows: (i) a ZnO:Ga MW was placed on a quartz substrate; (ii) the solution of AgNWs with certain concentration was deposited on the entire wire, and then annealed with vacuum heating (around 100 °C); (iii) increasing the volume of the droplets, AgNWs with controllable coverage concentration were conveniently deposited around the wires. Therefore, individual AgNWs@ZnO:Ga MWs were successfully prepared.

### 2.2 Device fabrication

A heterojunction device made of an individual ZnO:Ga MW and p-type Si crystal wafer was constructed. The fabricating process was briefly illustrated as follows: (i) Si crystal wafer was cleaned using trichloro ethylene, acetone, ethyl alcohol and deionized water, respectively; (ii) MgO nanofilm, working as the insulating layer, was evaporated on the one side of the Si crystal wafer through utilizing the electron-beam evaporation technique *via* a mask template. And the thickness of the evaporated MgO layer is about 100 nm. In the device architecture, the evaporated MgO layer was only used to avoid direct contact between the In electrode and Si crystal wafer, blocking carrier transportation from the In electrode to Si crystal wafer; (iii) Ni/Au electrode with a diameter of 2 mm was deposited on a Si crystal wafer through electron-beam evaporation system; (iv) an individual ZnO:Ga MW was put on a Si crystal wafer across the MgO nanofilm and Si crystal wafer, and fixed by an In particle on the MgO layer. In the device construction, Ni/Au and In were utilized as the electrodes for the current injection. Therefore, the heterostructure devices composed of Si crystal wafer and ZnO:Ga MW were fabricated.

### 2.3 Analysis methods

The surface morphology characterization of the as-prepared AgNWs, individual ZnO:Ga wires, and the wires decorated with AgNWs, were performed through scanning electron microscopy (SEM). The composition *via* elemental mapping of the individual ZnO:Ga MW was examined utilizing energy dispersive X-ray spectroscopy (EDS). The crystal structure of the wires was characterized using X-ray diffraction (XRD), with radiation source Cu-*K*_α1_ with *λ* = 1.540598 Å. AgNWs were further characterized utilizing transmission electron microscopy (TEM, JEOL-100CX). The optical properties of the AgNWs were tested using an ultraviolet-visible-near-infrared (UV-vis-NIR) spectrophotometer (Shimadzu 3600 plus). The electrical properties of an individual ZnO:Ga MW and fabricated n-ZnO:Ga MW/p-Si heterostructure were tested utilizing a Keysight semiconductor device analyzer (Keithley, B1500A). Photoluminescence (PL) of an individual ZnO:Ga MW with and without AgNWs was characterized with a He–Cd laser at wavelength 325 nm. EL characterization of the fabricated heterojunction was performed with a PIXIS 1024BR CCD detection system. While electrical measurements and photodetection characteristics of the single wire based photodetector were tested with a detection system containing a Xenon lamp, monochromator and semiconductor analysis device (Keithley B1500A). The light power density was corrected by a power meter. The time-resolved response was characterized with a detecting system containing a Xenon lamp, a chopper, a phase-locked amplifier and an oscilloscope. All the measurements were implemented at room temperature.

## 3 Results and discussion

### 3.1 Fabrication of n-ZnO:Ga MW/p-Si heterojunction LED

As previously reported, the as-grown ZnO:Ga wires with straight sidewalls, hexagonal cross-section and good crystallinity have been successfully fabricated through a one-step vapor transport deposition method.^22,38^ The optical picture of the synthesized samples is exhibited in Fig. 1(a). The lengths of the synthesized samples can reach up to 2.5 cm. The SEM image of a ZnO:Ga MW in Fig. 1(b) shows that the fabricated samples exhibit hexagonal cross-sectional and glossy facets. The corresponding EDS mapping of an individual ZnO:Ga MW was measured. Fig. 1(c) shows the uniform spatial distribution of Zn, O and Ga elements. The crystal structure of the as-grown ZnO:Ga MW was studied using XRD, as shown in Fig. 1(d). From the figure, three different diffraction peaks with 2*θ* values located at 31.74°, 34.39° and 56.58° can be obtained. These diffraction peaks correspond to (100), (002) and (110) planes, respectively, illustrating the hexagonal wurtzite structure of the as-synthesized ZnO:Ga MW. Additionally, the lattice constants *a* and *c* of the ZnO:Ga MW can be determined by utilizing the following equation:1
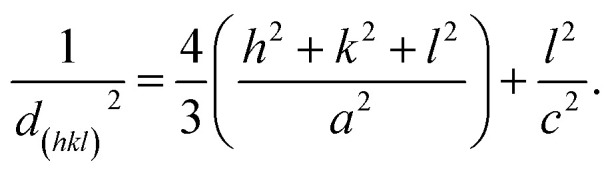
Therefore, the *a* and *c* values are calculated to be 3.25 Å and 5.21 Å, respectively. The XRD results indicate the good crystalline quality of the wire, and all the obtained Bragg reflections accord well with the Wurtzite *P*6_3_*mc* space.^41,42^Fig. 1(e) represents a typical TEM image of a single ZnO:Ga wire with orderly boundary and homogeneous width of about 350 nm. Further, the lattice fringes can be clearly noticed from the high-resolution TEM image obtained from the as-fabricated ZnO:Ga wire, as shown in Fig. 1(f). The interplanar distance of the sample is evaluated to be around 0.287 nm, which corresponds to the (100) lattice plane of the wurtzite ZnO monocrystals and matches well with the XRD results.^43,44^

AgNWs were introduced to cover an individual ZnO:Ga wire, the corresponding SEM image is displayed in Fig. 1(g). Clearly, the deposited AgNWs distribute randomly on the surface of ZnO:Ga MW. The optical characteristics of AgNWs have been measured using a UV-vis-NIR spectrometer. As shown in Fig. 1(h), the main wavelength of the extinction spectrum is positioned at around 370 nm. Thereby, the as-synthesized AgNWs show a plasmonic response in the ultraviolet wavelength.^35,39,45^ Besides, to describe the shape, size and uniformity of the as-synthesized AgNWs, TEM was conducted and the result is displayed in the inset of Fig. 1(h). It illustrates that the as-synthesized AgNWs exhibit a uniform size (the diameter is about 35 nm). Moreover, PL measurements for the bare wire and AgNWs decorated wire were conducted to research the influence of AgNWs on its optical characteristics. The PL spectrum for the bare ZnO:Ga wire is displayed in Fig. 1(i) (the violet solid line). Notably, the luminescence is dominated by ultraviolet light. And the ultraviolet emission centered at 378 nm reflects the typical near-band-edge (NBE) emission of the ZnO:Ga wire.^26,46^ Meanwhile, a negligible visible emission was also collected, which might originate from the Ga-doping induced impurity-related levels or intrinsic defects.^7,24^ By incorporating AgNWs, the ultraviolet light-emission has observably increased, and the enhanced ratio is extracted to about 3.8 times (see Fig. 1(i), the blue solid line). The increased ultraviolet emission could be contributed to by the introduced AgNWs. That is, upon ultraviolet illumination, the localized surface plasmon resonances of AgNWs can be excited, and the formed coupled interaction combined with adjacent ZnO:Ga MWs. As a consequence, the radiative recombination of the electron–hole around the ZnO:Ga MW-Si interface is improved with AgNWs deposition.^35,45,47^

**Fig. 2:**
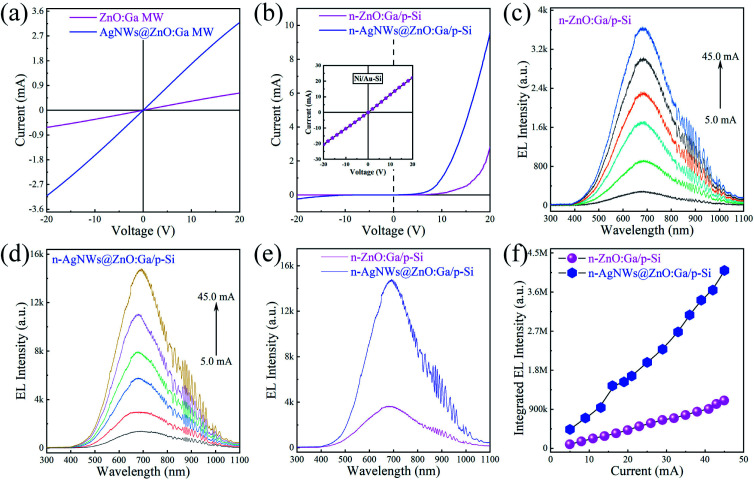
(a) *I*–*V* curves of single ZnO:Ga MWs deposited with and without AgNWs. (b) *I*–*V* curves of the individual ZnO:Ga wire based heterojunctions fabricated with and without AgNWs. Inset: *I*–*V* curve of p-Si substrate, with Ni/Au metals acting as the electrodes. (c) EL spectra obtained from the fabricated ZnO:Ga/Si heterostructure with the input current varying in the range of 5.0–45.0 mA. (d) EL spectra obtained from the fabricated AgNWs@ZnO:Ga MW based heterostructure upon different input current varying in the range of 5.0–45.0 mA. (e) EL spectra obtained from fabricated single MW based heterojunction devices, at the input current of 45.0 mA. (f) Integrated EL intensities of the fabricated heterojunction LEDs under different input currents.

The effect of the deposited AgNWs on the electronic transport characteristics of an individual ZnO:Ga wire were researched. Indium served as the electrode to fabricate In–ZnO:Ga–In structures (metal–semiconductor–metal). Electrical characterization of structures with and without AgNWs were performed. The current–voltage (*I*–*V*) curve of the structure displayed in Fig. 2(a) (the violet solid line) illustrates a linear feature, reflecting good ohmic contact between ZnO:Ga MW and In electrodes. By incorporating AgNWs, the electronic transport properties of the wire are significantly increased (see Fig. 2(a), the blue solid line).^24,38,46^ As we mentioned above, heterojunctions composed of a p-Si crystal wafer and an individual ZnO:Ga wire have incorporated Ni/Au and In as electrodes for carrier injection. Electrical characterization of the as-fabricated heterojunction devices was conducted. The *I*–*V* curve of the n-ZnO:Ga MW/p-Si heterojunction shown in Fig. 2(b) (the violet solid line) exhibits LED-like rectifying properties. Additionally, the *I*–*V* curve of p-type Si crystal wafer with Ni/Au electrode was characterized. The plotted *I*–*V* curve also demonstrates good ohmic contact, as shown in the inset of Fig. 2(b). Thus, a typical p–n junction between the p-Si and ZnO:Ga MW can be created. The turn-on voltage is evaluated as about 6.2 V. When operated over the turn-on voltage in a forward-biased regime, the fabricated n-ZnO:Ga MW/p-Si heterojunction structure can achieve red irradiation. The emitted photons were collected. We varied the input current from 5.0 to 45.0 mA, and the electroluminescence (EL) signals are displayed in Fig. 2(c). Clearly, the dominating EL peaks are located at ∼680 nm, originating from the depletion layer around the heterointerface of the ZnO:Ga wire and p-Si crystal wafer.^7,22^

To research the influence of AgNWs on the device performance, the same wire deposited with AgNWs was further applied to construct the device, and the *I*–*V* curve was measured, as exhibited in Fig. 2(b) (the blue solid line). Obviously, the electrical properties of the n-AgNWs@ZnO:Ga MW/p-Si heterostructure are observably enhanced. In particular, the contrast to a naked wire based heterostructure, we see that the input current of the AgNWs@ZnO:Ga MW LED is greatly increased up to about 10.0 mA at the forward bias of 20 V. And, the turn on voltage is significantly reduced to about 3.3 V. Furthermore, the lighting signals obtained from the heterostructure covered by AgNWs was plotted. The EL spectra depicted in Fig. 2(d) show that the main wavelengths of EL lighting emit at around 680 nm. To further demonstrate this enhancing phenomena, the EL signals obtained from fabricated single wire based devices at the same input current of 45 mA were collected, as shown in Fig. 2(e). This shows that a significantly enhanced ratio of about 4.0 times can be achieved. The integrated EL intensity of the fabricated LED *versus* input current were calculated. As shown in Fig. 2(f), the light output obtained from naked ZnO:Ga wire LED rises with the increase of the current, and observably increased EL irradiation can be acquired from the bare wire LED by incorporating AgNWs. Thus, profiting from the AgNWs, the light output from the as-fabricated LEDs increases significantly.^7,22,45^ As previous literature reported, the work function of the ZnO:Ga MW (∼4.6 eV) is a little higher than its electron affinity (∼4.35 eV).^48^ It can be concluded that the work function of Ag (∼4.26 eV) is lower than that of ZnO:Ga MW. As the forward bias is being turned on, the electrons in AgNWs will inject into ZnO:Ga MW, leading to an increased concentration of electrons in ZnO:Ga MW. As a consequence, the increased red-lighting is achieved, which profits from the incorporated AgNWs.

**Fig. 3:**
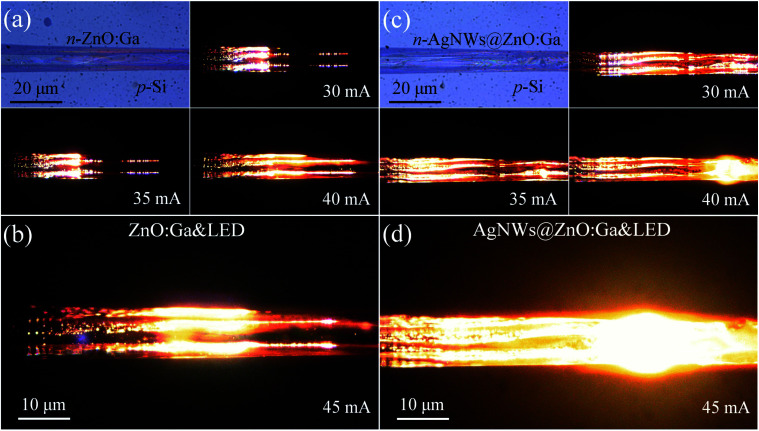
(a) Increasing the input current in the range of 35–40 mA, red luminescence can be observed from the fabricated n-ZnO:Ga MW/p-Si heterojunction device. The emission photos were taken using a microscopic digital camera. (b) An enlarged EL image was taken at the input current of 45 mA. (c) By incorporating AgNWs, the emission photos of the fabricated n-AgNWs@ZnO:Ga MW/p-Si heterojunction device were taken by varying the input current in the range 35–40 mA. (d) An enlarged EL image was taken at the input current of 45 mA.

The red light emitting from the electrically illuminated individual ZnO:Ga wire-based heterostructural LEDs was further characterized. The microscope EL images of the LEDs were collected using a CCD system in the dark field. The optical microscope photographs obtained from the fabricated n-ZnO:Ga MW/p-Si heterojunction LEDs are displayed in Fig. 3(a) under the injection current varying from 30 to 40 mA. The light irradiation can be noticed around nearly the whole wire, and the light output enhances significantly as the input current increases. The increased luminescence is also seen in Fig. 3(b) at the input current of 45 mA. After decorating with the AgNWs, an obviously improved light output and luminescence area of the prepared n-AgNWs@ZnO:Ga wire/p-Si heterojunction LED was achieved. The corresponding irradiation photographs are displayed in Fig. 3(c). In particular, brighter light illumination of the as-constructed heterojunction LED modified with AgNWs are observed, as shown in Fig. 3(d) (the input current of 45 mA).^7,22,49^ The influence of AgNWs on the light-emission properties is consistent with the increased emission intensity for the fabricated LEDs. Thus, the remarkably increased light-emission of the Si-based LEDs has been realized *via* AgNWs deposition.^7,22,50^

### 3.2 Fabrication of n-ZnO:Ga MW/p-Si photodetector

**Fig. 4:**
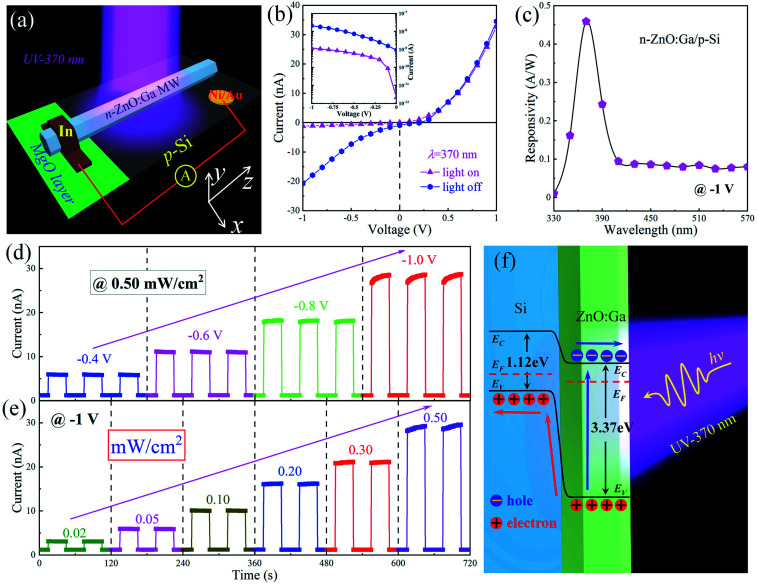
(a) Schematic diagram of the fabricated n-ZnO:Ga MW/p-Si heterostructure under 370 nm illumination. (b) *I*–*V* curves of n-ZnO:Ga MW/p-Si heterostructure in the dark, and under 370 nm illumination. Inset: logarithmic *I*–*V* curves in the reverse bias of −1 to 0 V. (c) The spectral responsivity of the fabricated n-ZnO:Ga MW/p-Si heterojunction device. (d) When exposed to 370 nm illumination with light intensity of 0.50 mW cm^−2^, the *I*–*t* curves of the fabricated n-ZnO:Ga MW/p-Si heterojunction device are formed by varying the applied bias from −1.0 to −0.4 V. (e) When operating at the reverse bias of −1 V, the *I*–*t* curves of the fabricated n-ZnO:Ga MW/p-Si heterostructure are formed under 370 nm illumination with light density in the range of 0.02–0.50 mW cm^−2^. (f) Schematic energy band diagram of the fabricated n-ZnO:Ga MW/p-Si heterostructure.

As we describe above, the prepared n-ZnO:Ga MW/p-Si heterostructure illustrates typical p–n rectifying characteristics, especially for the weak leakage current at the reverse bias range. When exposed to ultraviolet light at wavelength 370 nm, the fabricated heterojunction device exhibits a significant photoresponse at −1.0 V. The corresponding photodetecting characteristics of the fabricated n-ZnO:Ga MW/p-Si heterostructure are investigated. Fig. 4(a) exhibits the schematic architecture of the prepared n-ZnO:Ga MW/p-Si heterojunction photodetector, and the detailed fabrication process can be referred to in the Experimental section. *I*–*V* curves of the n-ZnO:Ga MW/p-Si heterojunction in the dark (the violet solid line) and under 370 nm light illumination with a light density of 0.50 mW cm^−2^ (the blue solid line) are shown in Fig. 4(b), and the logarithmic *I*–*V* curve is shown in the inset. The photocurrent is evaluated to be about 10^1^ to 10^2^ times higher than that of the dark current in the reverse bias regime.^2,9^

To evaluate the photoresponse performance of the detectors, responsivity (*R*), external quantum efficiency (EQE), linear dynamic range (LDR), and detectivity (D*) were studied and calculated.^51–53^ The responsivity is defined using the formula:2
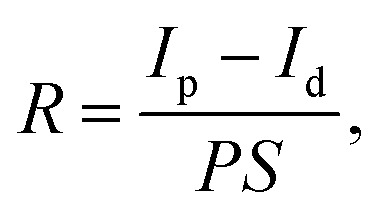
where *I*_p_ is the photocurrent, *I*_d_ is the dark current, *P* is the incident light intensity, *S* is the effective illuminated area. The EQE of the fabricated single MW heterojunction photodetector can be determined from the measured photocurrents *via* the following formula:3
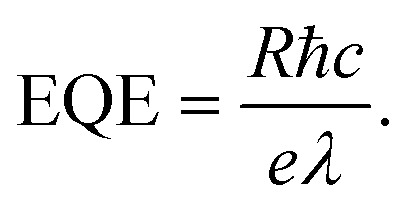
In the formula, ℏ is Planck’s constant, *c* is the speed of light in a vacuum, *e* is the electron charge, and λ is the wavelength of radiated light. The linear dynamic range of LDR is a figure-of-merit for the fabricated photodetector, and can be deduced as follows:4
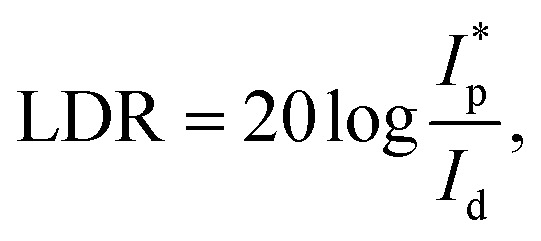
where 
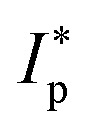
 is the photocurrent under ultraviolet illumination of 370 nm, with light intensity ∼ 0.50 mW cm^−2^. The detectivity D* of the single MW heterojunction device is used to express the ability to collect weak light signals, which is one of best features of these detectors. The D* can be calculated using the following formula:5
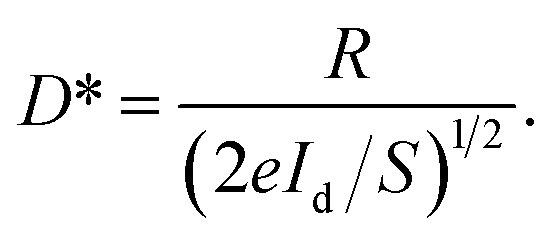


To find the working wavelength of the fabricated device, photoresponse in the spectral region varying from 330 to 570 nm was measured. Fig. 4(c) shows the responsivity as a function of light wavelength obtained from the heterojunction device.^54–56^ As observed, the detector displays an obvious photoresponse in the ultraviolet region. The strongest photoresponse peaks at 370 nm with the maximum responsivity of about 0.46 A W^−1^ at −1 V, which correspond well to the optical bandgap of ZnO:Ga MW (∼3.3 eV). Meanwhile, the relatively weak photoresponse in the visible light region might originate from the Si crystal wafer.^52,57^

The photoresponse of the fabricated n-ZnO:Ga MW/p-Si heterojunction photodetector (illuminated under 370 nm with light intensity of 0.50 mW cm^−2^) at voltages varying from −1.0 to −0.4 V were researched and summarized, as illustrated in Fig. 4(d). It is noticed that the photoresponse of the photodiode increases with photocurrent values: 6.15 nA at −0.4 V, 11.35 nA at −0.6 V, 18.32 nA at of −0.8 V, 28.50 nA of −1.0 V. The results correspond well with the fact that the photo-generation efficiency of the charge carriers is proportional to the applied voltages.^58,59^ Besides, the photoresponse of the fabricated single wire based heterojunction photodetector with light densities varying in the range 0.02–0.50 mW cm^−2^ at the reverse of −1 V were also studied, as shown in Fig. 4(e). Apparently, the photocurrent values increase from 3.32 nA at 0.02 mW cm^−2^, 5.91 nA at 0.05 mW cm^−2^, 10.18 nA at 0.10 mW cm^−2^, 15.88 nA at 0.20 mW cm^−2^, 21.27 nA at 0.20 mW cm^−2^, and finally to 29.17 nA at 0.50 mW cm^−2^, under 370 nm illumination. The photoresponse behaviors of the photodiode exhibit good reproducibility and stability in the reverse-biased regime. Furthermore, the results also correspond well to the fact that the photo-generation efficiency of the carriers is proportional to the light intensity.^60,61^

To understand the working mechanism of the photodetecting characteristics in the ultraviolet region, schematic diagrams of the n-ZnO:Ga MW/p-Si heterostructure energy bands and charge carrier transport are illustrated in Fig. 4(f). It shows that the large bandgap of ZnO:Ga MW make it a workable candidate to prepare ultraviolet photodetectors. When the photon energy of the illuminating light is higher than the bandgap of ZnO:Ga MW, the photon-generated electron–hole pairs are produced in the ZnO:Ga wire. As we described above, when the heterojunction between the n-ZnO:Ga MW and p-Si crystal wafer are formed, band discontinuity occurs around the ZnO:Ga/Si interface.^62,63^ Consequently, a classic type-II heterojunction is created in the n-ZnO:Ga MW/p-Si heterostructural configuration. With the carriers diffusing and moving in the ZnO:Ga wire and p-Si crystal wafer respectively, the band will bend and the depletion layer forms around the ZnO:Ga/Si interface. When the heterojunction is illuminated by ultraviolet illumination at wavelength ∼ 370 nm, the ground-state electrons in the valence band will jump into the conduction band in the ZnO:Ga wire. Meanwhile, profiting from the extra electric field and built-in field, the photon-generated carriers can be transferred along the semiconductors. And electrons will transport from the conduction band of the wire to the electrode, while holes are transported from the valence band of ZnO:Ga to the conduction band of p-Si.^52,57,58^ Thus, the responsivity in the ultraviolet band benefits from the photovoltaic influence of the fabricated n-ZnO:Ga MW/p-Si heterojunction.

The n-ZnO:Ga/p-Si heterojunction device also shows relatively poorer photodetection ability, including a responsivity of 0.46 A W^−1^, detectivity of 2.02 × 10^11^ Jones, photosensitivity (on/off ratio of about 10-fold), and a response speed of 596/403 μs, which we illustrate in the following. It is obviously shown that the bare n-ZnO:Ga MW/p-Si detector has no distinctly unique advantages on the ultraviolet photodetecting characteristics compared with previously reported ZnO/Si based photodetectors.^52,57,60^ The main problem to acquire high-performance Si-based photodetectors can be assigned to the indirect band gap, the insulating layer of SiO_*x*_, and the low carrier mobility of the Si-based materials and structures.^12^ Excitation of the localized surface plasmon resonances of nanostructured noble metals will result in strong resonance coupling and a distinguishable increase of the localized electromagnetic fields around nanoparticles. These phenomena have been generally investigated for plasmon-increased luminescence materials and devices, photodetecting devices, solar cells, surface enhanced Raman scattering and so on.^33,57,64^ In this regard, AgNWs with the desired plasmonic response in the ultraviolet region were further utilized to modulate the photodetecting performance of the fabricated n-ZnO:Ga MW/p-Si heterojunction photodetection devices.

**Fig. 5:**
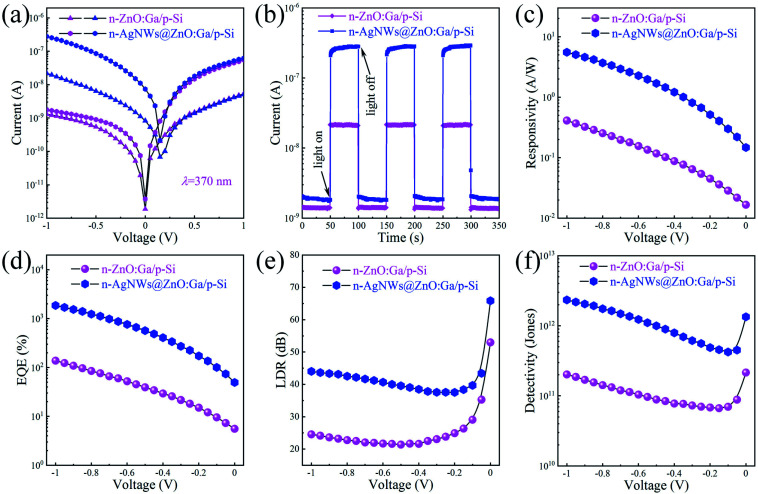
(a) Logarithmic *I*–*V* curves of the fabricated heterojunction devices under darkness and illumination of 370 nm, 0.50 mW cm^−2^. In the devices, an individual ZnO:Ga MW deposited with and without AgNWs was used. (b) Time-resolved photocurrent of the fabricated heterojunction devices with the ultraviolet light (370 nm, 0.50 mW cm^−2^) on and off at a voltage of −1 V. Comparison of the calculated responsivity (c), EQE (d), LDR (e) and detectivity (f) of the fabricated heterojunction devices, which are illuminated under ultraviolet light (370 nm, 0.50 mW cm^−2^) with a varied reverse bias from −1 to 0 V.

Fig. 5(a) presents the *I*–*V* curves of fabricated single MW heterostructural photodetectors, measured in the dark under 370 nm illumination (the light intensity ∼ 0.50 mW cm^−2^). In the heterojunction photodetector configuration, the used ZnO:Ga MW was not covered, and then covered with AgNWs. In darkness (the violet line), the fabricated heterojunction device with ZnO:Ga MW decorated by AgNWs exhibits a larger dark current than that of the naked ZnO:Ga wire based heterostructure, and the increased dark current is likely induced by the improved electron concentration in the wire decorated with AgNWs. As a contrast, the photocurrent of the AgNWs@ZnO:Ga wire based heterojunction photodetector can reach up to 278 nA at −1 V, which is much larger than that of the naked wire based heterojunction photodetector (∼22 nA).

Following the on/off light switching of the devices illuminated with a light intensity of 0.50 mW cm^−2^, the corresponding current–time (*I*–*t*) curves are plotted in Fig. 5(b). The on/off switching ratio of the fabricated n-ZnO:Ga MW/p-Si heterostructural photodetector is extracted to be about 10-fold in the ultraviolet band (the wavelength is 370 nm). By introducing AgNWs, the on/off switching ratio is increased up to 2.5 × 10^2^. The plotted *I*–*t* curves also exhibit good reproducibility and stability of the detectors. Thus, when operated upon identical conditions of applied bias and light intensity, the photocurrent of fabricated n-AgNWs@ZnO:Ga MW/p-Si heterostructural photodetector was found to be increased over one order of magnitude higher than that of the naked wire based photodetector. The introduction of AgNWs depositing on the ZnO:Ga wire displayed a strong contribution toward enhancing the photocurrent of the fabricated n-ZnO:Ga MW/p-Si heterostructural photodetector.^33,45,64^Fig. 5(c) illustrates the room-temperature photoresponse of the fabricated p–n heterojunction detectors with and without AgNWs deposition. The photodetection measurements were operated upon identical light intensity conditions. The peak responsivity of the as-constructed n-AgNWs@ZnO:Ga MW/p-Si heterostructural photodetector can reach up to 5.6 A W^−1^ at −1 V, which is significantly enhanced more than 11 times that of the bare wire based device (∼0.41 A W^−1^). By varying the adopted voltage from −1 to 0 V, AgNWs-enhanced photoresponse of the fabricated device is also observed.

According to eqn (3), the EQE of the fabricated n-AgNWs@ZnO:Ga MW/p-Si heterostructural photodetector is evaluated to be about 1.9 × 10^3^%, which is observably larger than that of the naked wire based heterojunction photodetector (∼145%). Fig. 5(d), shows the calculated EQE of the AgNWs@ZnO:Ga wire based heterojunction photodetector operated at different reverse bias, illustrating the significantly enhanced values. By incorporating AgNWs, the maximum LDR of the fabricated heterojunction photodetector is calculated to about 66 dB at the bias of 0 V. The LDR value is consistently larger than that of the naked ZnO:Ga wire based photodetector (∼53 dB), as shown in Fig. 5(e). By varying the reverse voltage, the LDR of the n-AgNWs@ZnO:Ga MW/p-Si heterojunction photodetector is consistently larger than that of the naked ZnO:Ga wire based device. The detectivities of the fabricated heterojunction photodetectors are displayed in Fig. 5(f). Profiting from the enhanced photoresponse of the photodetector covered by AgNWs, the detectivity can achieve 2.34 × 10^12^ Jones when measured under the reverse voltage of −1 V. The calculated D* is consistently higher than that of the naked ZnO:Ga wire based photodetector (2.02 × 10^11^ Jones). Conclusively, the incorporation of AgNWs with ultraviolet plasmonic properties can be used to enhance the photodetecting properties of the prepared n-ZnO:Ga MW/p-Si heterostructural photodetector.^57,59,64^

**Fig. 6:**
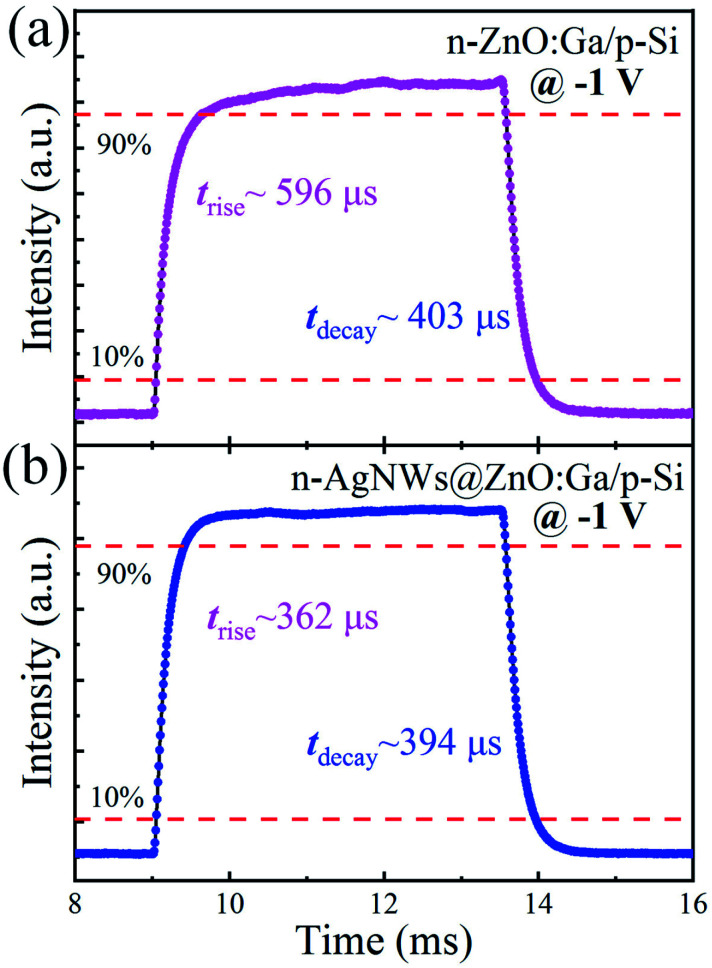
Time resolved photoresponse of the fabricated photodetectors, in which the device was constructed with (a) the naked ZnO:Ga wire and (b) the wire covered by AgNWs.

The time-resolved response of the fabricated photodetector was further tested. As shown in Fig. 6(a), when the n-ZnO:Ga MW/p-Si heterostructural device is illuminated under ultraviolet light at 370 nm, the rise and decay time is extracted to be about 596/403 μs. By introducing AgNWs, the obtained rise and decay time become ∼362/394 μs, significantly decreased compared with that of the naked wire based heterojunction detector, especially for the sharply reduced rise time (see Fig. 6(b)). Thereby, the enhancement of the response rate including the rising and falling edges is attributed to the deposition of AgNWs on the wire, resulting in coupling between AgNWs and ZnO:Ga MW in the fabricated photodetector.^21,50,64^ As the ultraviolet illumination turns on, the concentration of the photoinduced excitons increases drastically on account of the transformation of electrons from the valence band to the conduction band in the constructed n-ZnO:Ga MW/p-Si heterostructural photodetector. By introducing AgNWs, the excitation of localized plasmons of AgNWs can facilitate the generation of photo-induced carriers in the hybrid system. As a result, the response rate of the AgNWs decorated photodetector increases at the rising edge, which is dramatically faster than that of the naked wire-based device. When the ultraviolet light is switched off, the concentration of photo-induced carriers in the prepared AgNWs@ZnO:Ga MW/p-Si heterostructural photodetector decreases quickly, which results in a faster decrease of the current in contrast with that of the naked ZnO:Ga MW based device.^64,65^

**Fig. 7:**
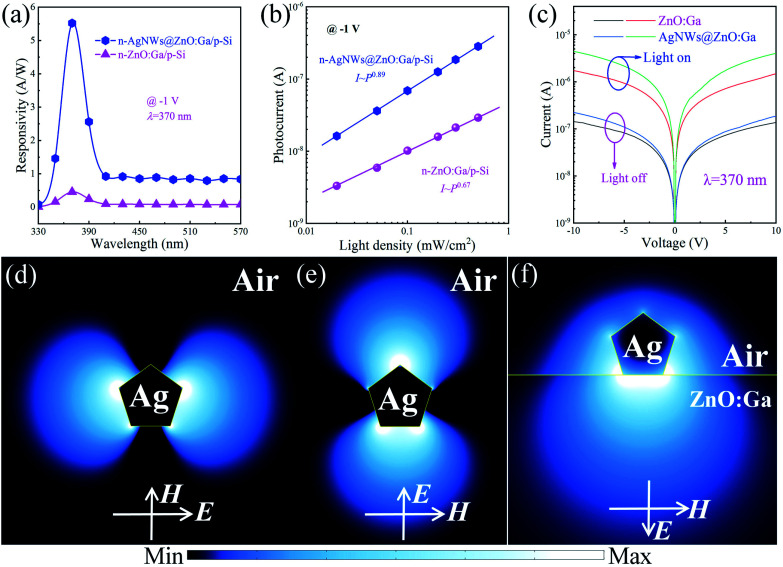
(a) Spectral responsivity curves of the fabricated heterojunction detectors under darkness and 370 nm irradiation with light density 0.50 mW cm^−2^. In the detector architecture, the used ZnO:Ga wire was not decorated, or decorated with AgNWs. (b) The photocurrents of the fabricated heterojunction devices as a function of the illuminated light intensity. (c) *I*–*V* characteristics of the ZnO:Ga wire not decorated, or decorated with AgNWs, under dark and ultraviolet light irradiation conditions (the incident wavelength was 370 nm, and the light intensity was 5.0 mW cm^−2^). The calculated electric-field distribution of an isolated AgNW obtained using FDTD simulation in the *x*–*y* plane, the light incidence direction and polarization along the (d) *x*-direction, and (e) *y*-direction. (f) Simulated electric-field distribution in the *x*–*y* plane of an isolated AgNW deposited on ZnO:Ga MW.

As mentioned above, the main extinction peak of the as-synthesized AgNWs locates at 370 nm, which matched well with the strongest responsivity peak of the fabricated photodetectors. Fig. 7(a) illustrates the spectral response of the fabricated single MW based heterojunction detectors, in which the ZnO:Ga MW was not decorated, or decorated with AgNWs. The detectors have been measured at voltage −1 V. The fabricated detectors not covered, or covered with AgNWs, both exhibit a distinct photoresponse in the ultraviolet region, with the maximum peak responsivities around 370 nm. The strongest peak responsivity of the n-AgNWs@ZnO:Ga MW/p-Si heterostructural detector can reach up to 5.52 A W^−1^ at 370 nm, which is observably increased to over 11 times that of the bare wire based detector (∼0.45 A W^−1^).^27,51^ To study the generation and recombination process of the fabricated photodetectors upon ultraviolet illumination at wavelength 370 nm, the photocurrents of the detectors not decorated, or decorated by AgNWs as a function of the light power were investigated. As exhibited in Fig. 7(b), the nonlinear relationship between the light power and photocurrent are defined with by ordinary power law:

6 *I*_p_ = *AP*^*θ*^.

In this formula, *A* is a constant with a certain wavelength, and the exponent *θ* (0.5 < *θ* < 1) illustrates the character on the generation and recombination behavior of the photocurrent. From eqn (6), the *θ* shows an ideal state of the devices, and can be influenced by the trap states. The *θ* value of the naked ZnO:Ga wire based heterojunction photodetector is calculated to be 0.68, indicating relatively worse junction character with many trap states in the detector. By incorporating AgNWs, the *θ* is increased up to 0.89. Thus, it can be inferred that the trap states decreased though AgNWs deposition, and the incident photons are more efficient for generating photocurrents in the fabricated photodetector. The increase of the power law factors suggests that the presence of AgNWs can result in resonant coupling between the excited ultraviolet plasmons of AgNWs and ZnO:Ga excitons.^60,66,67^

To study the optical response of the as-synthesized AgNWs, the photoconductive behavior of an individual ZnO:Ga wire prepared with and without AgNWs, was measured in the dark and in the presence of ultraviolet illumination (the incident wavelength is *λ* = 370.0 nm, and the light intensity is 5.0 mW cm^−2^). As shown in Fig. 7(c), *I*–*V* characteristics of the single ZnO:Ga wire not modified or modified with AgNWs, in darkness and under ultraviolet illuminated conditions, is found to be linear, suggesting the typical ohmic nature of the as-grown samples. In darkness, the single MW shows a linearly increasing current in the order of 10^−7^ A at the voltage of 10 V. By introducing AgNWs, the dark current of an individual ZnO:Ga MW exhibits little enhancement, which is in accordance with previously reported results.^35,45^ Compared with bare wire, this suggests that the increased or small variation of the dark current modulated by AgNWs, cannot be utilized to dominate the improvement of the photoresponse of the constructed n-ZnO:Ga MW/p-Si heterostructured detector. Illuminated under ultraviolet light, the photocurrents of the ZnO:Ga wire not modified, or modified by AgNWs, dramatically increases throughout the bias range. In particular, the photo to dark current ratio at −10 V is more than 22.5-fold for the ZnO:Ga MW covered by AgNWs, which is consistently larger than that of the bare wire based device (∼11.6).

The photodetecting abilities of the fabricated detector are significantly enhanced with the usage of AgNWs, and the increased characteristics can be assigned to the excited localized surface plasmon resonance of the deposited AgNWs on the wire. Because of the good overlap between the maximum extinction peak of the AgNWs and the strongest spectral response of the n-ZnO:Ga MW/p-Si heterojunction device, a coupling interaction between the AgNW plasmons and ZnO:Ga MW can occur. The plasmonic influence of the AgNWs on the devices were further investigated using theoretical simulation *via* a finite-different time-domain (FDTD) method. In the simulation, the complex relative permittivity of the AgNWs upon 370 nm light irradiation was modelled by experimental dielectric data, the refractive index of ZnO:Ga was 2.35, the refractive index of the air was 1.0, and the resonant wavelength was 370 nm.^7,38,45^ A three-dimensional model composed of a AgNW with a diameter of about 35 nm placed on ZnO:Ga MW was constructed. First, the spatial distributions of the electromagnetic field |*E*|^2^-intensity of a pentagon-shaped AgNW in the *x*–*y*-plane were investigated. As shown in Fig. 7(d) and (e), when the wavelength of irradiated illumination matched well with the resonance peak, the strongest intensity distribution of the localized electromagnetic field |*E*|^2^ is situated at the sharp tip of the pentagon-shaped cross section of the as-synthesized AgNWs. Second, the optical intense field concentration of the AgNW/ZnO:Ga hybrid structure in the *x*–*y*-plane was simulated. As demonstrated in Fig. 7(f), the obtained distribution of evanescent field intensity of the excited AgNW infiltrates into ZnO:Ga. This shows that the AgNWs-mediated electric field intensity can reduce sharply with the increase of distance between nanostructured materials and their neighboring medium (ZnO:Ga MW). As a result, the ultraviolet plasmonic response of the AgNWs induced the localization of electromagnetic energy around the AgNWs/ZnO:Ga interface, so is intensively reorganized. That is, upon ultraviolet excitation, the deposition of AgNWs on the MW leads to strong coupling of the AgNWs-plasmons and ZnO:Ga, resulting in the enhancement of light-detecting features for the ultraviolet light signals.^35,45,50^

The prepared n-ZnO:Ga MW/p-Si heterostructure exhibits ultraviolet photodetection, with negligible photo-detecting behavior in the visible region. By introducing AgNWs with the desired ultraviolet plasmonic response, the characteristics of the photodetector are further enhanced, containing a responsivity of 5.52 A W^−1^, detectivity of 2.34 × 10^12^ Jones, external quantum efficiency of 1.9 × 10^3^% upon 370 nm light irradiation at the reverse voltage of −1 V. Besides, the rise and fall times of AgNWs decorating the hybrid system are also significantly decreased, and are extracted to be 362 μs and 394 μs, respectively. All of the device parameters signified that the fabricated n-AgNWs@ZnO:Ga MW/p-Si heterostructural photodetector is highly workable for practical applications of ultraviolet photodetection. These enhanced photoresponse characteristics can be attributed to the absorption, originating from the excitation of localized plasmonic resonances of the deposited AgNWs. The proposed hybrid system made of the n-AgNWs@ZnO:Ga MW/p-Si heterojunction seems to be an alternative candidate for the development of high-performance ultraviolet photodetectors. To evaluate the characteristics of our fabricated ZnO:Ga/p-Si-based heterojunction photodetectors, we compare the devices with previous reported photodetectors based on various ZnO/Si-based materials and structures, which are summarized in Table 1. Considering that the fabricated n-AgNWs@ZnO:Ga MW/Si heterojunction photodetectors exhibit higher comprehensive performance and the advantage of simple manufacture, they are promising for future commercial applications. Specifically, the fabricated n-AgNWs@ZnO:Ga MW/p-Si heterojunction photodetector displays the fastest response time, which is the most competitive for practical applications. Besides, the responsivity of the AgNWs@ZnO:Ga MW-based detector is over one order of magnitude higher than the commercial product (DET10A2, THORLABS), as well as having a lower working voltage. Therefore, our findings stand out, especially for providing an inexpensive and suitable pathway for developing low-cost, miniaturized and integrated ultraviolet photodetectors.

**Table 1:** Comparison of the detection performance in this work and other related reports

Photodetector	Wavelength (nm)	Responsivity (A W^−1^)	Detectivity (Jones)	Rise/decay times	Ref.
AuNPs@ZnO/Si NWs	300–1100	1.0 (1 V)	∼2 × 10^11^	—	57
ZnO/NiO/Si film	∼260–350	∼10^2^ (8 V)	6.1 ×10^12^	14.8/9.7 s	52
ZnO/honeycomb-structured Si	365–1100	∼0.4 (2 V)	—	11/12 ms	60
ZnO/micro-Si	∼365–1100	0.14 (0.5 V)	—	—	62
ZnO/Si nanotubes	365	21.5 (2 V)	1.26 × 10^12^	0.44/0.59 s	63
ZnO NRs/Si	200–500	0.38 (4 V)	—	11/14 s	68
DET10A2 (THORLABS)	200–1100	0.44 (10 V)	—	1 ns/—	Commercial product
ZnO:Ga MW/Si	370	0.41 (−1 V)	2.02 × 10^11^	596/403 μs	This work
AgNWs@ZnO:Ga/Si	370	5.53 (−1 V)	2.34 ×10^12^	362/394 μs	This work

## 4 Conclusion

In summary, a smart and high performance luminescence and detecting bifunctional device based on a n-ZnO:Ga MW/p-Si heterostructure was demonstrated. At a forward-bias regime, visible-spectrum light-irradiation with the dominating wavelength peaking at around 680 nm was achieved. Meanwhile, the fabricated heterojunction can also exhibit a distinct ultraviolet photoresponse with the strongest photoresponsivity situated at 370 nm in the reverse-bias regime. To improve the opto-electrical performance of the fabricated heterojunction, AgNWs with uniform size, excellent electrical conductivity, and desired ultraviolet plasmonic properties were used to coat the fabricated n-ZnO:Ga MW/p-Si heterostructure. Profiting from the incorporated AgNWs, the red luminescence acquired from AgNWs@ZnO:Ga MW/p-Si heterojunction LED is considerably enhanced. Similarly, the AgNWs@ZnO:Ga MW heterojunction also exhibits better ultraviolet photoresponse properties compared with the bare wire-based detector. The increased characteristics of the detector reported in the present study can be regarded as even better than the performance parameters of previously published ZnO/Si-based materials and devices. The experimental findings provide a meaningful approach to developing low-dimensional, high-performance Si-based light emitting/detecting bifunctional optoelectronic devices.

## Conflicts of interest

There are no conflicts to declare.

## Supplementary Material

## References

[cit1] Liu H., Meng J., Zhang X., Chen Y., Yin Z., Wang D., Wang Y., You J., Gao M., Jin P. (2018). Nanoscale.

[cit2] Rein M., Favrod V., Hou C., Khudiyev T., Stolyarov A., Cox J., Chung C.-C., Chhav C., Ellis M., Joannopoulos J., Fink Y. (2018). Nature.

[cit3] Fadaly E., Dijkstra A., Suckert J., Ziss D., van Tilburg M., Mao C., Ren Y., van Lange V., Korzun K., Kölling S., Verheijen M., Busse D., Rödl C., Furthmüller J., Bechstedt F., Stangl J., Finley J., Botti S., Haverkort J., Bakkers E. (2020). Nature.

[cit4] Atabaki A. H., Moazeni S., Pavanello F., Gevorgyan H., Notaros J., Alloatti L., Wade M. T., Sun C., Kruger S. A., Meng H. (2018). Nature.

[cit5] Pan Z., Peng W., Li F., Cai Y., He Y. (2020). Adv. Funct. Mater..

[cit6] Lu Z., Hou G., Zhu Y., Chen J., Xu J., Chen K. (2021). Nanoscale.

[cit7] Liu Y., Jiang M., Tang K., Ma K., Wu Y., Ji J., Kan C. (2020). CrystEngComm.

[cit8] Liu L., Lyu J., Li T., Zhao T. (2016). Nanoscale.

[cit9] Bie Y. Q., Grosso G., Heuck M., Furchi M. M., Yuan C., Zheng J., Bunandar D., Navarro-Moratalla E., Lin Z., Efetov D. K. (2017). Nat. Nanotechnol..

[cit10] Cai Q., You H., Guo H., Wang J., Liu B., Xie Z., Chen D., Lu H., Zheng Y., Zhang R. (2021). Light: Sci. Appl..

[cit11] Mariotti D., Mitra S., Švrček V. (2013). Nanoscale.

[cit12] Li X., Chen M., Yu R., Zhang T., Song D., Liang R., Zhang Q., Cheng S., Dong L., Pan A., Wang Z. L., Zhu J., Pan C. (2015). Adv. Mater..

[cit13] Wang Z., Yu R., Wang X., Wu W., Wang Z. L. (2016). Adv. Mater..

[cit14] Shan Q., Wei C., Jiang Y., Song J., Zou Y., Xu L., Fang T., Wang T., Dong Y., Liu J., Han B., Zhang F., Chen J., Wang Y., Zeng H. (2020). Light: Sci. Appl..

[cit15] Deng Q., Wang Z., Wang S., Shao G. (2017). Sol. Energy.

[cit16] Baek S. H., Hasan M. R., Park I. K. (2016). Nanotechnology.

[cit17] Pyatkov F., Fuetterling V., Khasminskaya S., Flavel B., Hennrich F., Kappes M., Krupke R., Pernice W. (2016). Nat. Photonics.

[cit18] Birowosuto M. D., Yokoo A., Zhang G., Tateno K., Kuramochi E., Taniyama H., Takiguchi M., Notomi M. (2014). Nat. Mater..

[cit19] Fama S., Colace L., Gianlorenzo M., Assanto G., Luan H.-C. (2002). Appl. Phys. Lett..

[cit20] Dai W., Liu W., Yang J., Xu C., Alabastri A., Liu C., Nordlander P., Guan Z., Xu H. (2020). Light: Sci. Appl..

[cit21] Zhou J., Zhang J., Yang H., Wang Z., Shi J.-a., Zhou W., Jiang N., Xian G., Qi Q., Weng Y., Shen C., Cheng Z., He S. (2019). Nanoscale.

[cit22] Zhou X., Jiang M., Wu Y., Ma K., Liu Y., Wan P., Kan C., Shi D. (2020). Nanoscale Adv.

[cit23] Yadian B., Liu H., Wei Y., Wu J., Zhang S., Sun L., Zhao C., Liu Q., Ramanujan R. V., Zhou K., Gan C. L., Huang Y. (2014). Small.

[cit24] Liu Y., Jiang M., Zhang Z., Li B., Zhao H., Shan C., Shen D. (2018). Nanoscale.

[cit25] Zhang Z., Ning Y., Fang X. (2019). J. Mater. Chem. C.

[cit26] You J. B., Zhang X. W., Zhang S. G., Tan H. R., Ying J., Yin Z. G., Zhu Q. S., Chu P. K. (2010). J. Appl. Phys..

[cit27] Dong J., Wang Z., Wang X., Wang Z. L. (2019). Nano Today.

[cit28] Minotto A., Haigh P. A., Lukasiewicz L. G., Lunedei E., Gryko D. T., Darwazeh I., Cacialli F. (2020). Light: Sci. Appl..

[cit29] Ye X., Zheng C., Chen J., Gao Y., Murray C. B. (2013). Nano Lett..

[cit30] Sun L., Zhang D., Sun Y., Wang S., Cai J. (2018). Adv. Funct. Mater..

[cit31] Chatterjee D., Shetty S., Müller-Caspary K., Grieb T., Krause F. F., Schowalter M., Rosenauer A., Ravishankar N. (2018). Nano Lett..

[cit32] Liu K., Sakurai M., Liao M., Aono M. (2010). J. Phys. Chem. C.

[cit33] Dai M., Chen H., Feng R., Feng W., Hu Y., Yang H., Liu G., Chen X., Zhang J., Xu C.-Y., Hu P. (2018). ACS Nano.

[cit34] An Q., Meng X., Sun P. (2015). ACS Appl. Mater. Interfaces.

[cit35] Yang L., Wang Y., Xu H., Liu W., Zhang C., Wang C., Wang Z., Ma J., Liu Y. (2018). ACS Appl. Mater. Interfaces.

[cit36] Wu Y., Hasan T., Li X., Xu P., Wang Y., Shen X., Liu X., Yang Q. (2015). Appl. Phys. Lett..

[cit37] Wang D., Allcca A. E. L., Chung T.-F., Kildishev A. V., Chen Y. P., Boltasseva A., Shalaev V. M. (2020). Light: Sci. Appl..

[cit38] Sun Z., Jiang M., Mao W., Kan C., Shan C., Shen D. (2020). Photonics Res.

[cit39] Zhu X., Xu J., Qin F., Yan Z., Guo A., Kan C. (2020). Nanoscale.

[cit40] Zhu X., Guo A., Xu J., Kan C. (2020). CrystEngComm.

[cit41] Shunmuga Sundaram P., Sangeetha T., Rajakarthihan S., Vijayalaksmi R., Elangovan A., Arivazhagan G. (2020). Physica B Condens. Matter.

[cit42] Lu J., Xu C., Li F., Yang Z., Peng Y., Li X., Que M., Pan C., Wang Z. L. (2018). ACS Nano.

[cit43] Luan C., Vaneski A., Susha A., Xu X., Wang H.-E., Chen X., Xu J., Zhang W., Lee C.-S., Rogach A., Zapien J. A. (2011). Nanoscale Res. Lett..

[cit44] Liu W., Xiu F., Sun K., Xie Y.-H., Wang K. L., Wang Y., Zou J., Yang Z., Liu J. (2010). J. Am. Chem. Soc..

[cit45] Wu Y., Xu J., Jiang M., Zhou X., Wan P., Kan C. (2020). CrystEngComm.

[cit46] Zhao B., Jiang M.-M., Zhao D.-X., Li Y., Wang F., Shen D.-Z. (2015). Nanoscale.

[cit47] Wang X., Liu K., Chen X., Li B., Jiang M., Zhang Z., Zhao H., Shen D. (2017). ACS Appl. Mater. Interfaces.

[cit48] Quemener V., Alnes M., Vines L., Rauwel P., Nilsen O., Fjellvåg H., Monakhov E. V., Svensson B. G. (2012). J. Phys. D: Appl. Phys..

[cit49] Yang X., Shan C.-X., Ni P.-N., Jiang M.-M., Chen A.-Q., Zhu H., Zang J.-H., Lu Y.-J., Shen D.-Z. (2018). Nanoscale.

[cit50] Yang L., Liu W., Xu H., Ma J., Zhang C., Liu C., Wang Z., Liu Y. (2017). J. Mater. Chem. C.

[cit51] Zhao B., Wang F., Chen H., Wang Y., Jiang M., Fang X., Zhao D. (2015). Nano Lett..

[cit52] Hwang J.-D., Liao C.-Y. (2020). J. Phys. Chem. C.

[cit53] Su L., Ouyang W., Fang X. (2021). J. Semicond..

[cit54] Wang H., Kim D. H. (2017). Chem. Soc. Rev..

[cit55] Zhao B., Wang F., Chen H., Zheng L., Su L., Zhao D., Fang X. (2017). Adv. Funct. Mater..

[cit56] Shin D. H., Ko J. S., Kang S. K., Choi S.-H. (2020). ACS Appl. Mater. Interfaces.

[cit57] Samanta C., Bhattacharya S., Raychaudhuri A. K., Ghosh B. (2020). J. Phys. Chem. C.

[cit58] Zhou H., Gui P., Yang L., Ye C., Xue M., Mei J., Song Z., Wang H. (2017). New J. Chem..

[cit59] Thongma S., Tantisantisom K., Grisdanurak N., Boonkoom T. (2019). Sens. Actuator A Phys..

[cit60] Lim S., Um D.-S., Ha M., Zhang Q., Lee Y., Lin Y., Fan Z., Ko H. (2017). Nano Res..

[cit61] Goswami L., Aggarwal N., Krishna S., Singh M., Vashishtha P., Singh S. P., Husale S., Pandey R., Gupta G. (2020). ACS Omega.

[cit62] Chatzigiannakis G., Jaros A., Leturcq R., Jungclaus J., Voss T., Gardelis S., Kandyla M. (2020). ACS Appl. Electron. Mater..

[cit63] Flemban T. H., Haque M. A., Ajia I., Alwadai N., Mitra S., Wu T., Roqan I. S. (2017). ACS Appl. Mater. Interfaces.

[cit64] Ouyang W., Teng F., Jiang M., Fang X. (2017). Small.

[cit65] Jin Z., Zhou Q., Chen Y., Mao P., Li H., Liu H., Wang J., Li Y. (2016). Adv. Mater..

[cit66] Kind H., Yan H., Messer B., Law M., Yang P. (2002). Adv. Mater..

[cit67] Zeng L.-H., Wang M.-Z., Hu H., Nie B., Yu Y.-Q., Wu C.-Y., Wang L., Hu J.-G., Xie C., Liang F.-X., Luo L.-B. (2013). ACS Appl. Mater. Interfaces.

[cit68] Al-Hardan N., Jalar A., Abdul Hamid M., Keng L. K., Ahmed N., Shamsudin R. (2014). Sens. Actuator A Phys..

